# Unexpected Imidazole Coordination to the Dirhodium Center in a Protein Environment: Insights from X-ray Crystallography and Quantum Chemistry

**DOI:** 10.1021/acs.inorgchem.2c01370

**Published:** 2022-05-24

**Authors:** Domenico Loreto, Francesca Fasulo, Ana B. Muñoz-García, Michele Pavone, Antonello Merlino

**Affiliations:** ‡ Department of Chemical Sciences, University of Naples Federico II, Via Cintia, Napoli I-80126, Italy; § Department of Physics “Ettore Pancini”, University of Naples Federico II, Via Cintia, Napoli I-80126, Italy

## Abstract

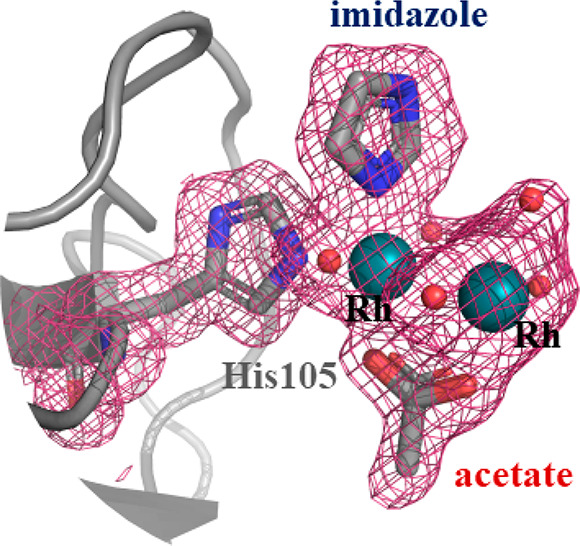

X-ray diffraction data demonstrate that the adduct formed upon the reaction of dirhodium(II,II) tetraacetate with RNase A reacts with imidazole, leading to the formation of an unexpected product with the imidazole that binds the dirhodium center at an equatorial site rather than an axial site. The origin of this result has been dissected using quantum-chemical calculations.

## Abstract 2

The dirhodium(II,II) tetraacetate/RNase A adduct reacts with imidazole, leading to the formation of an unexpected product with the imidazole that binds the dirhodium center at an equatorial site rather than an axial site. The origin of this result has been dissected using quantum-chemical calculations.

Dirhodium(II) complexes with bridging equatorial ligands, such as [Rh_2_(μ-O_2_CR)_4_]L_2_ (R = Me, Et, Pr; L = axially coordinated solvent), are cytotoxic compounds and efficient catalysts for a number of reactions,^1−7^ including hydrogen evolution and CO_2_ reduction.^8−10^ It is generally accepted that the reaction of [Rh_2_(μ-O_2_CCH_3_)_4_] with nitrogen-containing ligands (L_N_) produces axially substituted [Rh_2_(μ-O_2_CCH_3_)_4_](L_N_)_2_ species.^11−13^ In particular, when L_N_ is imidazole (Im), the [Rh_2_(μ-O_2_CCH_3_)_4_](Im)_2_ species forms; the X-ray structure of this species has been reported.^13^

We have recently proven by several experimental techniques, including X-ray crystallography, that [Rh_2_(μ-O_2_CCH_3_)_4_] reacts with the model protein bovine pancreatic ribonuclease (RNase A), forming adducts with the side chains of His105 and His119 that coordinate the dirhodium center at the axial site.^14,15^ In these adducts, over time, the acetate ligands are mostly replaced by water molecules.^14,15^ With the aim of verifying the dirhodium compound reactivity upon formation of the adduct with the protein at the crystal state, here we solved the X-ray structure of the reaction product of the dirhodium tetraacetate/RNase A system with Im. This represents a rare example of *in crystallo* reactivity of a metal/protein adduct.

Crystals of the adduct formed upon the reaction of dirhodium tetraacetate with RNase A were prepared according to the previously reported procedure.^14^ These crystals were then treated with a solution of Im dissolved in the reservoir. X-ray diffraction data on these crystals were collected at the XRD2 beamline of Elettra synchrotron, Trieste, Italy. Data collection and refinement statistics are reported in Table S1. The structure, which was solved at 1.40 Å resolution and refined to a *R* factor of 18.9 (*R*-free 23.1), contains two molecules in the asymmetric unit (molecules A and B; Figure S1). The final model was deposited in the Protein Data Bank under the accession code 7QHR. The two molecules in the asymmetric unit are very similar to each other (root-mean-square deviation, rmsd, between equivalent carbon α atoms is as low as 0.39 Å) and to those in the crystal of the adduct of the protein with dirhodium tetraacetate (rmsd = 0.22 and 0.15 Å for molecules A and B, respectively).

As expected, the dirhodium center is found close to the side chains of His105 and His119 in both molecules A and B (Figures S2–S4 and 1). Close to the dirhodium core bound to the side chain of His119 of molecules A and B, three acetate ions and two water molecules are found at the equatorial dirhodium sites, while a water molecule is found at the axial site (Figures S2 and S3). Analysis of the electron density maps reveals that, close to the dirhodium centers coordinated to the side chains of His105 of the two protein molecules in the asymmetric unit, an Im ligand is found. Interpretation of the electron density map in molecule A is complicated by the presence of alternative conformations of the dirhodium-containing fragment (Figure S4). However, in molecule B, this site is very well-defined. Surprisingly, Im does not bind the dirhodium center at the axial coordination site, as expected, but it coordinates one of the two rhodium atoms of the dimetallic core at the equatorial site (Figure 1). The Im binding to the dirhodium center is stabilized by a network of hydrogen bonds and contacts that the heterocycle compound forms with surrounding residues and solvent molecules (Figure 1). The (di)rhodium coordination sphere is completed by water molecules and an acetate ligand (Figure 1).

**Figure 1:**
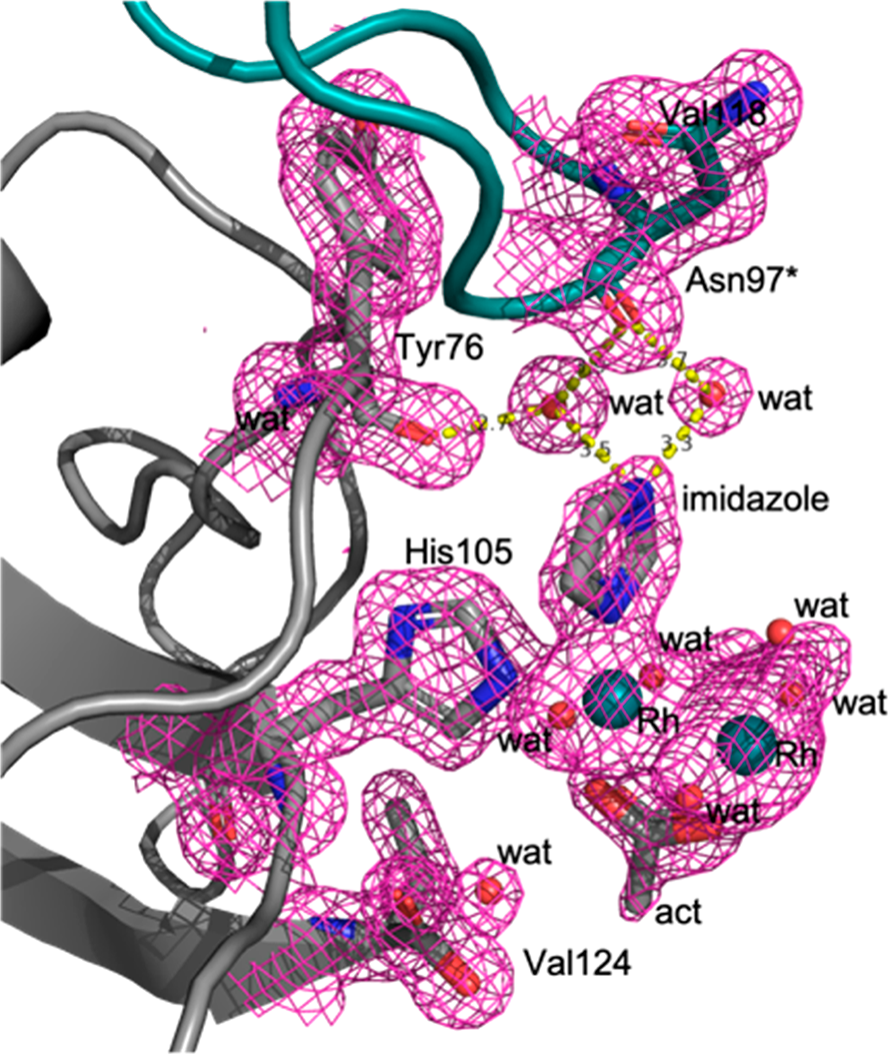
Im binding site in the structure of RNase A treated with [Rh_2_(μ-O_2_CCH_3_)_4_] and then with Im. The dirhodium center is coordinated to the side chain of His105. The 2*F*_o_ – *F*_c_ electron density map is reported at 1.0 σ and colored in pink.

To shed light on the origin of this unexpected experimental evidence, we investigated the reactivity and selectivity of the observed dirhodium tetraacetate/protein adduct (at His105) with a second Im by quantum chemistry,^17−19^ within the framework of density functional theory (DFT; see the computational details in the Supporting Information).

Following the cluster approach recently reviewed by Himo and co-workers,^20,21^ we carved from the crystal structure a small portion of the dirhodium/protein adduct around the His105 residue (for cluster model, see Figure 2a). We built this model based on simple considerations on the distance of the surrounding residues from the dirhodium core. We also applied, for a comparison, a simplified minimal model where the protein environment is neglected, and we only considered an Im molecule at the axial position, mimicking the coordination of His105. In both cases, we also used implicit solvation via the polarizable continuum model to account for the dielectric properties of the chemical environment. We selected water as the solvent because the dirhodium adduct is exposed to aqueous solution and it is almost fully hydrolyzed. For both models, we characterized coordination of the second Im molecule to the three available sites: the axial site opposite to His105/Im (ax), equatorial to the second rhodium atom (r2) and equatorial to the rhodium atom that is already coordinated by His105/Im (r), as depicted by Figure 2.

**Figure 2:**
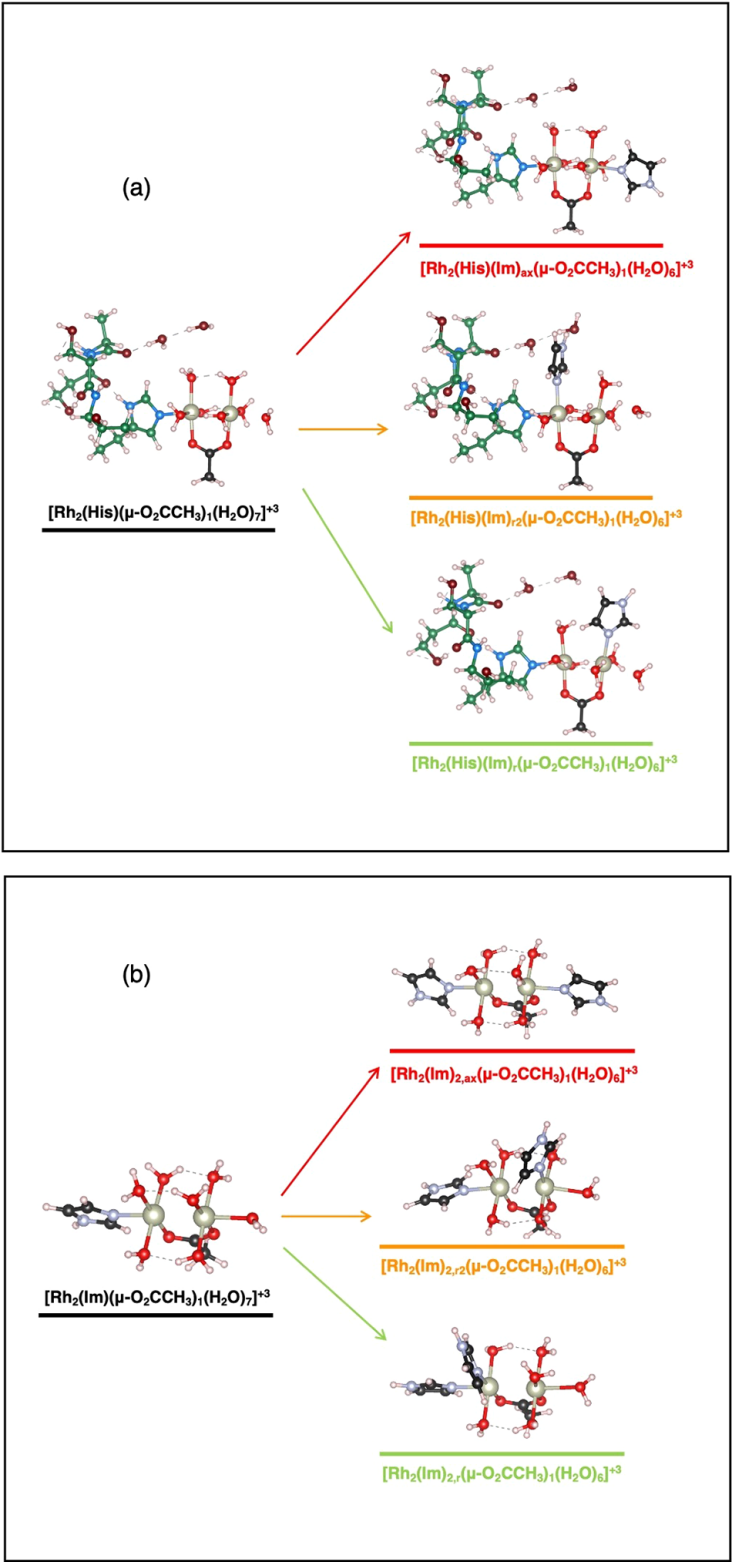
Substitution reactions with inclusion of the second Im molecule for the dirhodium/protein adduct (cluster model) (a) and for the corresponding minimal model (b). Legend: Rh, silver; O, red; N, light blue; C, green/black; H, white.

The free energies for all of these substitutions were computed according to eq 1 and are listed in Table 1.
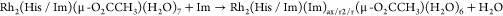
1

**Table 1:** Free-Energy Variations (Δ*G*) upon Substitution of a Water Molecule (eq 1) with Im at Different Coordination Sites and Relative Free Energy Variations (Δ*G*_rel_) with Respect to the Experimentally Found Equatorial Site (r)

		ax	r2	r
Δ*G* (eV)	cluster model	–0.398	–1.004	–1.173
	minimal model	–0.482	–0.940	–1.003
Δ*G*_rel_ (eV)	cluster model	0.776	0.169	0.0
	minimal model	0.544	0.081	0.0

As clearly highlighted by the computational data, there is a specific preference for Im coordination to the equatorial site (r), in agreement with experiments. Comparing the cluster and minimal models, we can find that the presence of the surrounding protein residues improves the thermodynamic driving force for coordination at the (r) site with respect to the other two options. However, we must note that the minimal model is indeed able to catch the correct binding trend, and so it can be used further to rationalize this process.

First, we considered the formation of hydrated species, where six water molecules replaced three tetraacetate groups, as found in the protein environment. We found that the cost of removing the three ligands is not balanced by coordination of the water molecules, and the free energy variation upon going from [Rh_2_(Im)(μ-O_2_CCH_3_)_4_(H_2_O)] to [Rh_2_(Im)(μ-O_2_CCH_3_)(H_2_O)_7_] is positive (i.e., unfavorable) with a value of ∼5 eV (Figure S5). Our calculations considered that the acetate groups enter into the solvent as isolated moieties. The protein environment, evidently, balanced the thermodynamics for this process by providing convenient coordination spots for the leaving acetate groups, and concurrently it also provided extra stabilization via the second-shell hydrogen-bond network. From the hydrated species [Rh_2_(Im)(μ-O_2_CCH_3_)(H_2_O)_7_], where the axial Im molecule is modeling the His105 side chain, the second Im molecule favorably substitutes the water molecule at the (r) equatorial site, in agreement with the experiments.

The reason behind such unexpected Im binding can be found in the electronic features of the [Rh_2_(Im)(μ-O_2_CCH_3_)(H_2_O)_7_] compound, where the two rhodium atoms are not equivalent because of the presence of the Im(His105) ligand, as depicted by Figure 3. Bader’s Atom-in-Molecule charge analysis of the starting compounds (Figure 3, top panel) shows that the rhodium bound to Im(His) is slightly more positive than the other rhodium, and thus it can act as a better Lewis acid site for the second Im.

**Figure 3:**
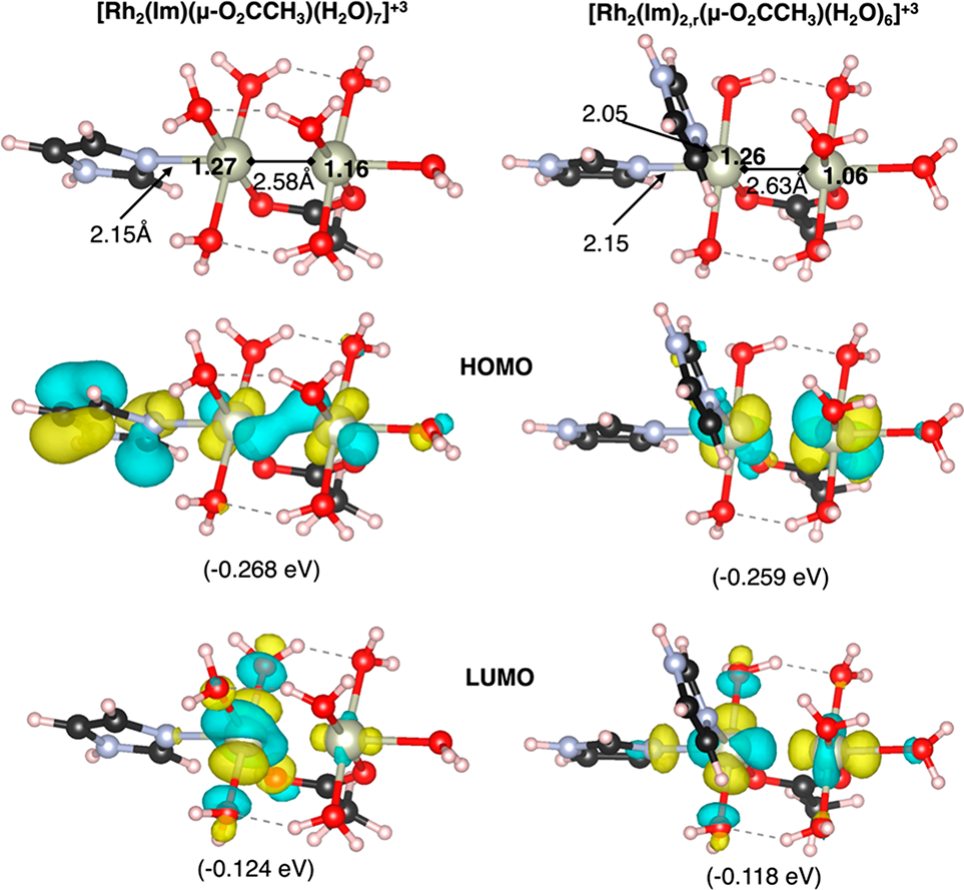
Top panel: Computed Bader charges on rhodium atoms and selected distances for [Rh_2_(Im)(μ-O_2_CCH_3_)(H_2_O)_7_] and [Rh_2_(Im)_2,r_(μ-O_2_CCH_3_)(H_2_O)_6_]. Central (bottom) panel: HOMO (LUMO) of both adducts and corresponding energies. Color code as in Figure 2, in which the isodensity surfaces are depicted as yellow and cyan for positive and negative values, respectively. The distances and Bader’s effective charges are reported for rhodium in the top panel, and the HOMO and LUMO energies are reported in parentheses.

It is worth noting that the degree of hydrolysis also plays a noninnocent role in the observed and computed selectivity. We calculated the substitution free energies for a water molecule by Im on different sites also for the [Rh_2_(Im)(μ-O_2_CCH_3_)_4_(H_2_O)], [Rh_2_(Im)(μ-O_2_CCH_3_)_3_(H_2_O)_3_], and [Rh_2_(Im)(μ-O_2_CCH_3_)_2_(H_2_O)_5_] series, i.e., for adducts with four, three, and two acetate ligands, to be compared to the highly hydrolyzed [Rh_2_(Im)(μ-O_2_CCH_3_)(H_2_O)_7_] considered above. We found that configurations with the second Im at the axial position are by far the least favorable for all hydrolysis degrees (Figure S5). However, regarding the equatorial positions, (Im)_2,r2_-like configurations are slightly preferred over (Im)_2,r_ ones for adducts with three and two acetate groups. The experimentally obtained (Im)_2,r_ one becomes the most stable only for the adduct with one acetate group. Of course, the presence of a second-shell hydrogen-bonding network with protein residues can alter these relative energies. However, it is already proven that the protein itself has the key role of sufficiently hydrolyzing the tetraacetate compound,^14−16^ so that when Im is added, the final (Im)_2,r_ is formed.

The new dirhodium complex has peculiar electronic features that can result in modified (photo)catalytic activity, as shown by the qualitative differences in computed frontier orbitals of [Rh_2_(Im)_2,r_(μ-O_2_CCH_3_)(H_2_O)_6_] with respect to [Rh_2_(Im)(μ-O_2_CCH_3_)(H_2_O)_7_] (Figure 3). For example, the involvement in the highest occupied molecular orbital (HOMO) of the Im ligand π system in [Rh_2_(Im)(μ-O_2_CCH_3_)(H_2_O)_7_] is lost in [Rh_2_(Im)_2,r_(μ-O_2_CCH_3_)(H_2_O)_6_], and the lowest unoccupied molecular orbital (LUMO) presents a more significant contribution from the second rhodium than in the single Im complex (Figure 3). Further experimental and theoretical characterizations of the photophysical and/or catalytic properties of this new dirhodium center are ongoing in our laboratory.

In conclusion, we characterized from the structural point of view the product of the reaction of a dirhodium/protein adduct with Im. Interestingly, at solid state the dirhodium complex reacts with Im, forming an unexpected product, with Im bound at the equatorial position. This result can have important implications considering that [Rh_2_(μ-O_2_CCH_3_)_4_] binds human serum albumin, the most abundant plasma protein, through coordination to histidine residues,^13^ and that the binding deeply affects the metal compound efficacy as an anticancer agent. The reaction mechanism was elucidated by quantum-chemical calculations. DFT results predict the same coordination complex as in experiments to be the most stable once the original dirhodium complex undergoes hydrolyzation during formation of the adduct with the protein. The origin of the observed unexpected preference toward coordination of the Im moiety to the equatorial position is unveiled by electronic structure analysis, which also highlights peculiar features of frontier molecular orbitals. Our combined experimental and theoretical results pave the way to further investigations of this unprecedented protein-bound dirhodium center, opening at the same time new directions in the exploitation of the crystal reactivity toward new coordination complexes of transition-metal cations.

## Supporting Information Available

Experimental section, crystallography, DFT, Figures S1–S5, and Tables S1 and S2 (PDF)
