# Supplementary material for: Unexpected Imidazole Coordination to the Dirhodium Center in a Protein Environment: Insights from X-ray Crystallography and Quantum Chemistry
Source: Inorg Chem. 2022 May 24;61(22):8402–5. doi: 10.1021/acs.inorgchem.2c01370 (PMC9175176; doi:10.1021/acs.inorgchem.2c01370)
Supplement: Supplementary file 1 — ic2c01370_si_001.pdf [file ic2c01370_si_001.pdf]

Supporting Information

Unexpected imidazole coordination to dirhodium center in a protein environment: insights from X-ray crystallography and quantum chemistry

Domenico Loreto,[1,‡] Francesca Fasulo, [1,‡] Ana B. Muñoz-García,[2] Michele Pavone, [1,*] Antonello Merlino[1,*]

1 Department of Chemical Sciences, University of Naples Federico II, Via Cintia, I-80126, Napoli, Italy

2 Department of Physics "Ettore Pancini", University of Naples Federico II, Via Cintia, I-80126, Napoli, Italy

‡ These authors equally contributed to this work

* Corresponding authors: michele.pavone@unina.it ; antonello.merlino@unina.it

Experimental section

Crystallography

RNase A crystals were grown at 298 K using the hanging drop vapor diffusion method as previously reported,[1] using protein concentration of 20 mg mL^{-1} and a reservoir consisting of 22 % PEG 4K, 10 mM sodium citrate pH 5.1. To study the Im reactivity with the adduct formed upon reaction of [Rh_2(μ-O_2CCH_3)$_4$] with RNase A, crystals of RNase A were soaked with a 10 mM solution of [Rh_2(μ-O_2CCH_3)$_4$] for ten days. These crystals were then soaked with a saturated solution of Im for 44 days. X-ray diffraction data were measured on these crystals at the XRD2 beamline of Elettra synchrotron in Trieste, Italy. Data sets were processed and scaled through global phasing AutoPROC pipeline.[2] Data collection statistics are reported in Table S1. The crystal structure was solved by molecular replacement with Phaser[3] using the PDB code 1JVT (molecule A)[4] as a search model. Restrained refinements were carried out using Refmac5.[5] Coot was used for the visualization of the electron density map and for model building.[6] Refinement statistics are reported in Table S1. Figures were prepared using Pymol.[7]

Density functional theory calculations

DFT calculations were performed with Gaussian16.[8] The TZVP basis set was used for C, N, H and O atoms and the SDD effective core potential (ECP) and basis set for Rh.

Structural optimizations, molecular frequencies, and thermochemistry data were obtained at the B3LYP[9,10] level of theory, including Grimme's dispersion correction D3 with the Becke-Johnson damping function.[11,12] The water solvent is considered with the polarizable continuum model (PCM).[13] We tested also the SMD[14] model of implicit solvation on selected structures and compare it to PCM as listed in Table S2, but we found no significant differences with PCM and we kept this last model.

The default maximum force and displacement tolerance parameters in Gaussian were considered for the ground-state minimum-energy structures. Molecular frequencies were computed within the harmonic oscillator approximation, and the thermochemical data were computed at room temperature (298 K).

References

1 Vitagliano, L.; Merlino, A.; Zagari, A.; Mazzarella, L.; - Productive and nonproductive binding to ribonuclease A: X-ray structure of two complexes with uridylyl(2',5')guanosine. Protein Sci. 2000, 6, 1217-1225.

2 Vonrhein, C.; Flensburg, C.; Keller, P.; Sharff, A.; Smart, O. Paciorek, W.; Womack, T.; Bricogne, G.; - Data Processing and Analysis with the autoPROC Toolbox. Acta. Cryst. D. 2011, 67, 293-302.

3 McCoy, A. J.; Grosse-Kunstleve, R. W.; Adams, P. D.; Winn, M. D.; Storoni, L. C.; Read.,

R. J.; - Phaser Crystallographic Software. J. Appl. Cryst. 2007, 40, 658-674.

4 Vitagliano, L.; Merlino, A.; Zagari, A.; Mazzarella. L.; - Reversible Substrate-induced Domain Motions in Ribonuclease A. Proteins 2002, 46, 97-104.

5 Murshudov, G. N.; Vagin, A. A.; Dodson., E. J.; - Refinement of Macromolecular Structures by the Maximum-likehood Method. Acta. Cryst. D. 1997, 53, 240-255.

6 Emsley, P.; Cowtan, K.; - Coot: Model-building Tools for Molecular Graphics. Acta. Cryst. D. Biol. Cryst. 2004, 60, 2126-2132.

7 DeLano. W. L.; - The PyMOL Molecular Graphics System. 2002.

8 Frisch, M. J. ; Trucks, G. W.; Schlegel, H. B.; Scuseria, G. E.; Robb, M. A.; Cheeseman, J. R.; Scalmani, G.; Barone, V.; Petersson, G. A.; Nakatsuji, H.; Li, X.; Caricato, M.; Marenich, A. V.; Bloino, J.; Janesko, B. G.; Gomperts, R.; Mennucci, B.; Hratchian, H. P.; Ortiz, J. V.; Izmaylov, A. F.; Sonnenberg, J. L.; Williams-Young, D.; Ding, F.; Lipparini, F.; Egidi, F.; Goings, J.; Peng, B.; Petrone, A.; Henderson, T.; Ranasinghe, D.; Zakrzewski, V. G.; Gao, J.; Rega, N.; Zheng, G.; Liang, W.; Hada, M.; Ehara, M.; Toyota, K.; Fukuda, R.; Hasegawa, J.; Ishida, M.; Nakajima, T.; Honda, Y.; Kitao, O.; Nakai, H.; Vreven, T.; Throssell, K.; Montgomery, J. A. Jr., Peralta, J. E.; Ogliaro, F.; Bearpark, M. J.; Heyd, J. J.; Brothers, E. N.; Kudin, K. N.; Staroverov, V. N.; Keith, T. A.; Kobayashi, R.; Normand, J.; Raghavachari, K.; Rendell, A. P.; Burant, J. C.; Iyengar, S. S.; Tomasi, J.; Cossi, M. Millam, J. M.; Klene, M. Adamo, C.; Cammi, R.; Ochterski, J. W.; Martin, R. L.; Morokuma, K.; Farkas, O.; Foresman, J. B.; Fox. D. J.; -Gaussian 16. 2016.

9 Becke., A. D.; A new mixing of Hartree–Fock and local density-functional theories J. Chem. Phys. 1993, 98, 1372.

10 Lee, C.; Yang, W.; Parr., R. G.; Development of the Colle-Salvetti correlation-energy formula into a functional of the electron density. Phys. Rev. B 1988, 37, 785-789.

11 Becke, A. D.; Johnson, E. R.; - A Simple Effective Potential for Exchange. J. Chem. Phys. 2006, 124, 221101.

12 Grimme, S.; Antony, J.; Ehrlich, S.; Krieg., H.; - A Consistent and Accurate Ab Initio Parametrization of Density Functional Dispersion Correction (DFT-D) for the 94 elements H-Pu. J. Chem. Phys. 2010, 132, 154104.

13 Tomasi, J.; Mennucci, B.; Cammi., R.; - Quantum Mechanical Contiuum Solvation Models. Chem. Rev. 2005, 105, 2999-3093.

14 Marenich, A. V.; Cramer, C. J.; Truhlar, D. G. Universal solvation model based on solute electron density and on a continuum model of the solvent defined by the bulk dielectric constant and atomic surface tensions. J. Phys. Chem. B 2009, 113, 6378-6396.

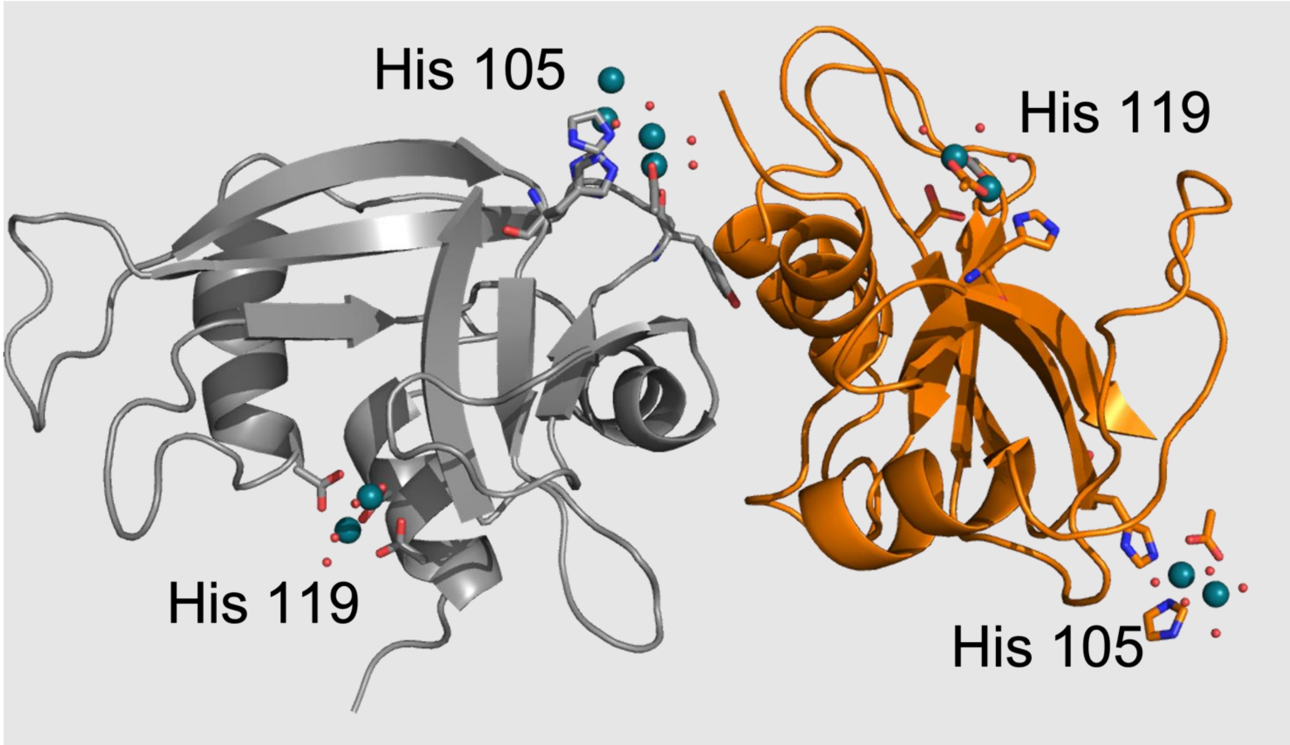

Figure S1. Overall structure of crystals of RNase A treated with $[Rh_2(\mu\text{-}O_2CCH_3)_4]$ and then with Im. The two molecules in the asymmetric unit are colored in grey and orange.

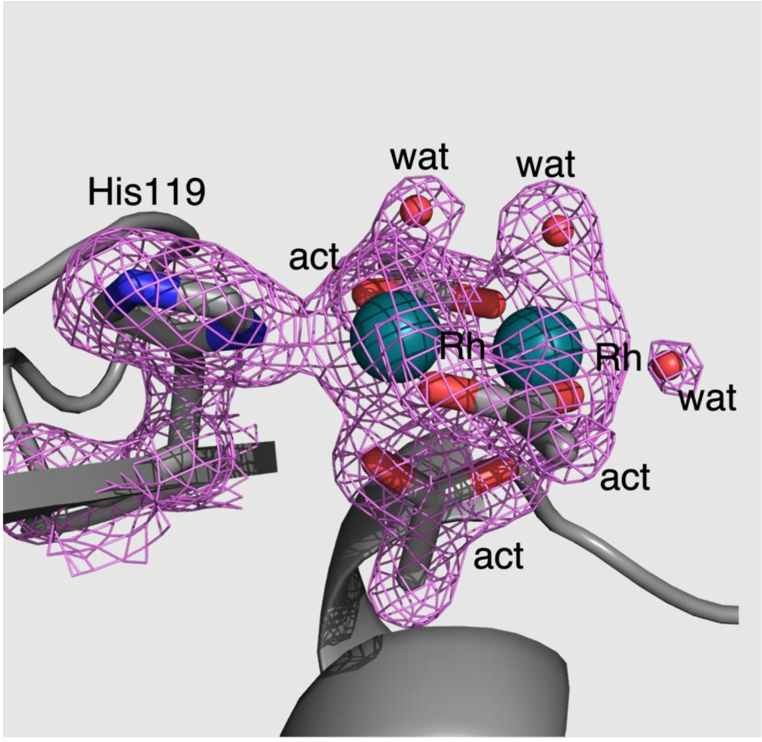

Figure S2. Details of the Rh binding site close to the side chain of His119 in molecule A of the crystal of RNase A soaked with a solution of $[Rh_2(\mu\text{-}O_2CCH_3)_4]$ and then with Im. The side chain of His119 coordinates at the axial coordination site. 2Fo−Fc electron density maps are contoured at 1.0σ (pink).

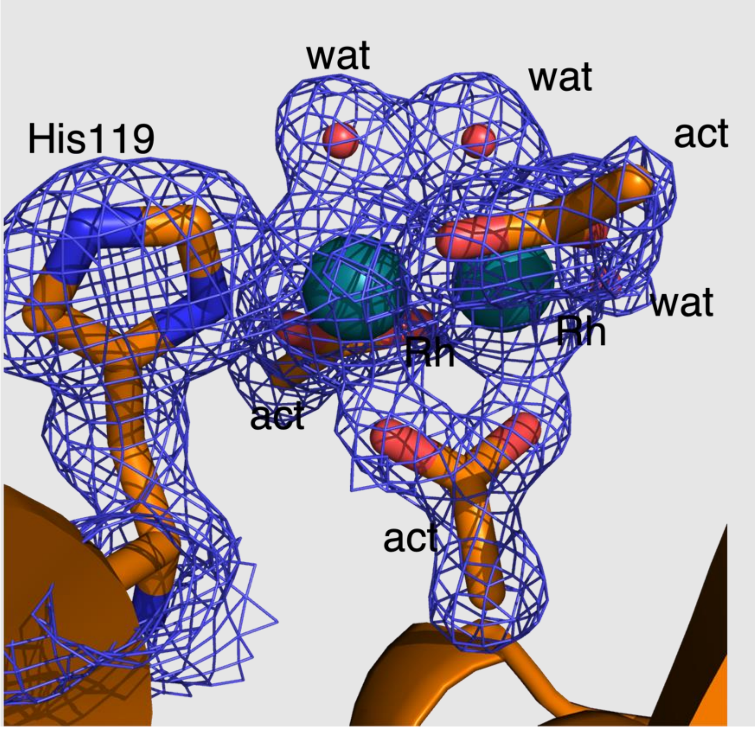

Figure S3. Details of the Rh binding site close to the side chain of His119 in molecule B of the crystal of RNase A soaked with a solution of [$Rh_2(\mu\text{-}O_2CCH_3)_4$] and then with Im. The side chain of His119 coordinates at the axial coordination site. 2Fo−Fc electron density maps are contoured at 1.0σ (blue).

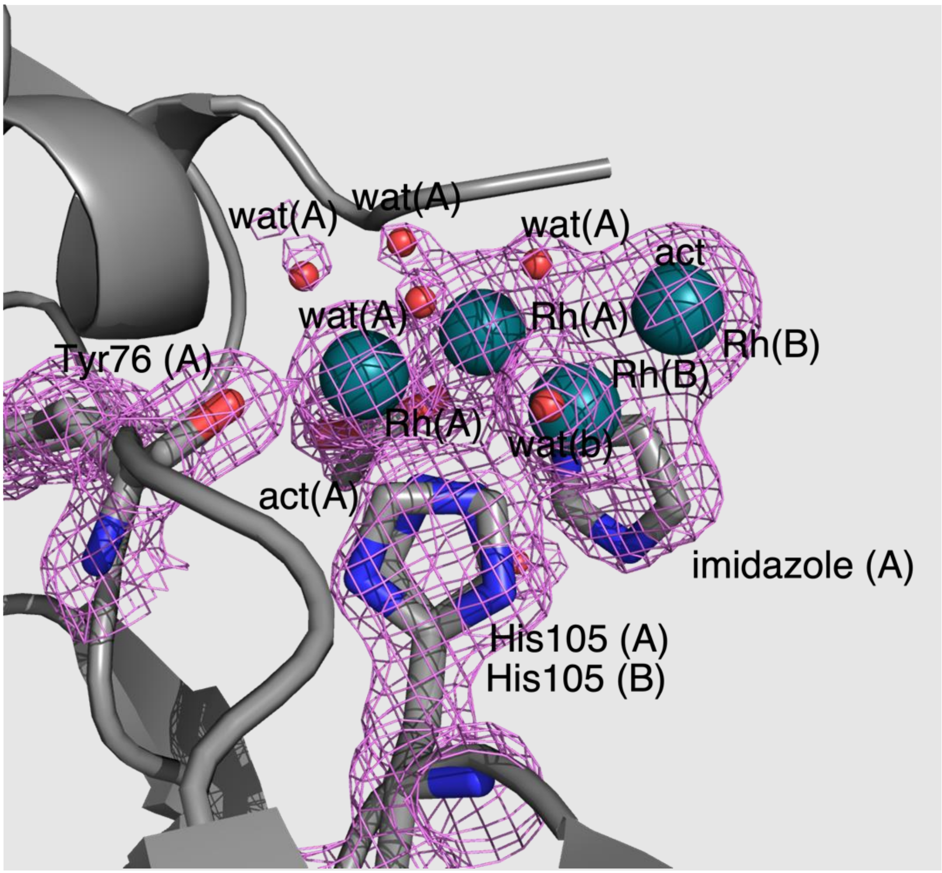

Figure S4. Details of the Rh binding site close to the side chain of His105 in molecule A of the crystal of RNase A soaked with a solution of [$Rh_2(\mu\text{-}O_2CCH_3)_4$] and then with Im. In this site, the interpretation of the 2Fo-Fc electron density map (contoured at 1.0σ and colored in pink) is complicated by the presence of an alternative position of the dirhodium fragment coupled to a flip of His105 side chain.

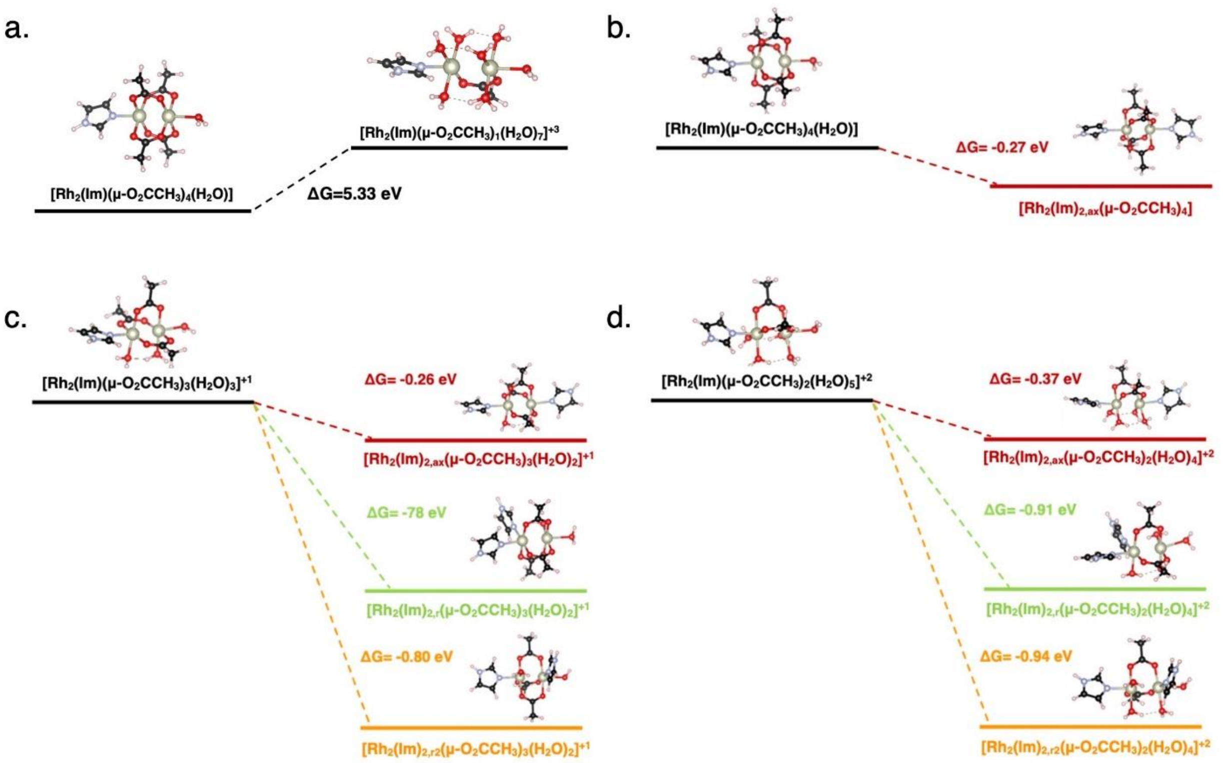

Figure S5. The substitution free energies for water molecules by three μ-O_2CCH_3 (a) and Im on different sites. All possible adducts with four (b), three (c) and two (d) acetate ligands are considered.

Table S1. Data collection and refinement statistics for the crystal of RNase A soaked with a solution of [$Rh_2(\mu\text{-}O_2CCH_3)_4$] and then with Im

Space group	C2
Unit-cell parameters	
a (Å)	100.500
b (Å)	32.530
c (Å)	72.200
β (°)	90.30
Resolution limits (Å)	50.3-1.40 (1.43-1.40)
No. of unique reflections	43550
No. of observations	280144
Completeness (%)	94.3 (92.5)
Rmeas	0.058 (1.079)
I/σ(I)	15.4 (2.2)
Average multiplicity	6.4 (6.8)
CC half	1.0 (0.8)
a.u. content	Two protein molecules
Refinement	
Resolution (Å)	50.30-1.40
No. of reflections	41609
Rfactor/Rfree (%)	18.9/23.1
No. of atoms	2323
Overall B factors ($Å^2$)	24.9
Rh occupancy	0.80/0.80/0.70/0.70/0.55/0.55/0.50/0.50/0.50/0.50
Rh B-factors ($Å^2$)	24.8/30.5/25.0/37.4/20.0/25.0/25.4/23.1/20.6/53.3
R.m.s. deviations	
Bond lengths (Å)	0.014
Bond angles (°)	3.95
Ramachandran plot (%)	
Favored	97.1
Allowed	1.9
Disallowed	0.9
PDB code	7QHR

Table S2. Substitution free energy variations for the DiRh-Im complex computed with PCM and SMD solvent models (see eq. 1 and Table 1 in main text).

		$(Im)_{2,ax}$	$(Im)_{2,r2}$	$(Im)_{2,r}$
ΔG_{REL} (eV)	PCM	0.544	0.081	0.000
	SMD	0.519	0.076	0.000
ΔG (eV)	PCM	-0.482	-0.940	-1.030
	SMD	-0.159	-0.603	-0.679
